# Influenza A(H3N2) Outbreak on a University Campus — Michigan, October–November 2021

**DOI:** 10.15585/mmwr.mm7049e1

**Published:** 2021-12-10

**Authors:** Miranda J. Delahoy, Lindsey Mortenson, Laura Bauman, Juan Marquez, Natasha Bagdasarian, Joseph Coyle, Kelsey Sumner, Nathaniel M. Lewis, Adam S. Lauring, Brendan Flannery, Manish M. Patel, Emily T. Martin

**Affiliations:** ^1^Epidemic Intelligence Service, CDC; ^2^Influenza Division, National Center for Immunization and Respiratory Diseases, CDC; ^3^University of Michigan University Health Service, Ann Arbor, Michigan; ^4^Washtenaw County Health Department, Ypsilanti, Michigan; ^5^Michigan Department of Health and Human Services; ^6^University of Michigan School of Medicine, Ann Arbor, Michigan; ^7^University of Michigan School of Public Health, Ann Arbor, Michigan.

On November 10, 2021, the Michigan Department of Health and Human Services (MDHHS) was notified of a rapid increase in influenza A(H3N2) cases by the University Health Service (UHS) at the University of Michigan in Ann Arbor. Because this outbreak represented some of the first substantial influenza activity during the COVID-19 pandemic, CDC, in collaboration with the university, MDHHS, and local partners conducted an investigation to characterize and help control the outbreak. Beginning August 1, 2021, persons with COVID-19–like* or influenza-like illness evaluated at UHS received testing for SARS-CoV-2, influenza, and respiratory syncytial viruses by rapid multiplex molecular assay.^†^ During October 6–November 19, a total of 745 laboratory-confirmed influenza cases were identified.^§^ Demographic information, genetic characterization of viruses, and influenza vaccination history data were reviewed. This activity was conducted consistent with applicable federal law and CDC policy.^¶^

During October 6–November 19, among 3,121 persons tested, 745 (23.9%) received a virus test result that was positive for influenza A, 137 (4.4%) for SARS-CoV-2, and 84 (2.7%) for respiratory syncytial virus. Overall, >95% of influenza cases were detected during November 1–19 (Figure), suggesting rapid spread. One patient with confirmed influenza A infection was hospitalized. Among patients with positive influenza test results, the median age was 19 years (range = 17–31 years), 54.1% were female, 60.0% resided off-campus, 34.6% resided in on-campus residence halls, and 5.4% resided in fraternity or sorority houses. Among 380 specimens sequenced for influenza, all viruses belonged to the A(H3N2) 2a.2 subgroup, which diversified recently from the influenza A(H3N2) subclade 3C.2a1b.2a viruses (i.e., full clade: 3C.2a1b.2a.2). Among 2,405 persons who received testing for influenza A during October 6–November 12, 128 of 481 persons (26.6%) with positive influenza test results and 512 of 1,924 persons (26.6%) with negative influenza test results had documented receipt of 2021–22 influenza vaccine ≥14 days before the test.**

**FIGURE:**
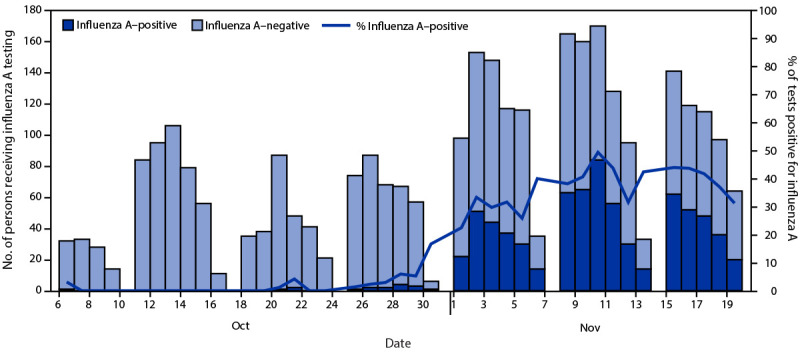
Number of symptomatic persons who received testing for influenza A at University Health Service (N = 3,121)* and percentage of tests positive for influenza A, by date of influenza test^†^ — University of Michigan, October 6–November 19, 2021 * Among persons who received testing more than once during October 6–November 19, 2021, the first influenza A–positive test result was used, or if the person never received an influenza A–positive result, the first negative test result was used. ^†^ University Health Service does not conduct influenza A testing on Sundays.

Available influenza vaccines are designed to provide protection against four different influenza viruses: A(H1N1)pdm09, A(H3N2), B/Victoria lineage, and B/Yamagata lineage. Historically, vaccine effectiveness has been lower against influenza A(H3N2) viruses than against influenza A(H1N1)pdm09 or influenza B viruses, likely because A(H3N2) viruses evolve more rapidly and are able to escape immunity (*1*). The A(H3N2) component of the northern hemisphere 2021–22 influenza vaccines was updated in February 2021 to protect against a newly emerging 3C.2a1b.2a subclade, which now includes two subgroups (2a.1 and 2a.2) (*2*). The 2a.2 subgroup of H3N2 viruses detected in Michigan is genetically related to, but antigenically distinguishable (i.e., lower postinfection ferret antibody cross-reactivity) from 2a.1-like H3N2 virus included in the northern hemisphere 2021–22 influenza vaccines (*3*). The similar vaccination rates among persons with positive and negative influenza test results in this outbreak suggest that protection against mild infection with the 2a.2 subgroup of H3N2 viruses was low among these mostly younger adults. However, cautious interpretation of this finding is needed for reasons such as the potential for incomplete vaccination history and changing coverage with ongoing vaccination campaigns. Persons included in this analysis had mild influenza illness, and vaccination offers protection against a spectrum of outcomes such as hospitalization and death, which occur rarely and are difficult to measure in this age group (*4*). Results for this specific 2a.2 subgroup of H3N2 viruses are not generalizable to other age groups, populations at higher risk, or other influenza viruses that might circulate. Additional investigation and monitoring are needed to determine vaccine effectiveness against circulating H3N2 viruses in other settings, in other groups of persons, and against other influenza viruses that might emerge this season.

The findings of this investigation highlight the importance of increasing vigilance for influenza disease this winter, as indicated in CDC’s Health Alert Network Health Advisory issued on November 24, 2021 (*5*). Given the substantial impact of COVID-19 on health care systems, with a weekly rate of approximately 500 or more COVID-19 cases per 100,000 population in Michigan during the week ending November 19, 2021 (*6*), additional strategies to reduce influenza illness are important. Several measures can help mitigate severe influenza and the resulting strain on health care services. First, improving influenza vaccination coverage in persons aged ≥6 months, particularly those who are at higher risk for serious influenza complications, is critical to reducing influenza-associated illnesses, hospitalizations, and deaths. Compared with influenza vaccination coverage in 2020, coverage is lower so far this season in certain groups at higher risk for severe influenza illness, such as pregnant persons and children. Second, clinicians should consider diagnostic testing for influenza and SARS-CoV-2 infection for patients with acute respiratory illness, especially among hospitalized patients and those at higher risk for complications. Third, treatment with influenza antiviral medications can reduce influenza complications and should be used in all patients with suspected or diagnosed influenza who are hospitalized, in outpatients who develop progressive disease, and in outpatients with increased risk for complications (*7*). Influenza antivirals also can be used to reduce the risk for influenza among asymptomatic persons who have been exposed to someone who has influenza (i.e., postexposure prophylaxis) (*7*). Influenza antivirals have historically been used for postexposure prophylaxis among residents in institutional settings, such as long-term care facilities, to help control influenza outbreaks. In the context of ongoing COVID-19 surges, influenza antiviral treatment and prophylaxis could also be considered for persons living in other communal settings (e.g., shelters, university residence halls, or prisons) to reduce strain on health care services in these institutions during influenza outbreaks. Fourth, nonpharmaceutical interventions that are used for prevention of COVID-19, such as physical distancing, masking, routine surface cleaning, hand hygiene, and proper cough etiquette, might also provide protection against influenza (*8*). To help mitigate the potential severity of the influenza season, public health practitioners and clinicians should recommend and offer the current seasonal influenza vaccine to all eligible persons aged ≥6 months.

## Footnotes

* Signs and symptoms consistent with COVID-19–like illness include fever or chills, cough, shortness of breath or difficulty breathing, fatigue, muscle or body aches, headache, recent loss of taste or smell, sore throat, congestion or runny nose, nausea or vomiting, or diarrhea.

† GeneXpert (Cepheid).

§ October 6, 2021, was the date of the first confirmed influenza A case among persons with COVID-19–like or influenza-like illness who visited UHS since August 2021.

¶ 45 C.F.R. part 46, 21 C.F.R. part 56; 42 U.S.C. Sect. 241(d); 5 U.S.C. Sect. 552a; 44 U.S.C. Sect. 3501 et seq.

** Persons with documented receipt of 2021–22 influenza vaccination in the UHS record or Michigan Care Improvement Registry who had been vaccinated ≥14 days before the influenza test date were considered vaccinated. Persons without a documented 2021–22 influenza vaccination in the UHS record or Michigan Care Improvement Registry were considered unvaccinated. Persons with a documented 2021–22 influenza vaccination in the UHS record or Michigan Care Improvement Registry who had been vaccinated <14 days before the influenza test date were excluded. A total of 2,405 persons tested for influenza A during October 6–November 12 were considered vaccinated or unvaccinated, after the exclusion of persons vaccinated <14 days before the influenza test date. Vaccination data are subject to lag; therefore, an earlier cutoff was used for reporting of vaccination status compared with that for confirmed influenza A cases.
